# A group III patatin-like phospholipase gene *pPLAIIIδ* regulates lignin biosynthesis and influences the rate of seed germination in *Arabidopsis thaliana*

**DOI:** 10.3389/fpls.2023.1212979

**Published:** 2023-07-13

**Authors:** David Charles Simiyu, Jin Hoon Jang, Ok Ran Lee

**Affiliations:** ^1^ Department of Applied Plant Science, College of Agriculture and Life Science, Chonnam National University, Gwangju, Republic of Korea; ^2^ Interdisciplinary Program in IT-Bio Convergence System, Chonnam National University, Gwangju, Republic of Korea; ^3^ Botany Department, College of Natural and Applied Sciences, University of Dar es Salaam, Dar es Salaam, Tanzania

**Keywords:** phospholipases, lignin, chlorophylls, pPLAIIIδ, MGDG

## Abstract

The lignification of plant secondary walls is an important process that provides plants with mechanical support. However, the presence of lignin in the secondary walls affects the readily availability of cellulose required in various industries, including the biofuel, paper, and textile industries. Thus, plants with less lignin are ideal for usage in such industries. Molecular studies have identified genes that regulate plant lignification, including group III plant-specific patatin-related phospholipase genes. Recent studies have reported decreased lignin content when *pPLAIIIα, pPLAIIIγ* (from *Arabidopsis thaliana*), and *pPLAIIIβ* (from *Panax ginseng*) were overexpressed in *Arabidopsis*. However, the role played by a closely related gene *pPLAIIIδ* in lignin biosynthesis has not yet been reported. In this study, we found that overexpression of the *pPLAIIIδ* significantly reduced the lignin content in secondary cell walls, whereas the silencing of the gene increased secondary walls lignification. Transcript level analysis showed that the key structural and regulatory genes involved in the lignin biosynthesis pathway decreased in overexpression, and increased in plants with silenced *pPLAIIIδ*. Further analysis revealed that *pPLAIIIδ* played an influential role in several physiological processes including seed germination, and chlorophyll accumulation. Moreover, the gene also influenced the size of plants and plant organs, including leaves, seeds, and root hairs. Generally, our study provides important insights toward the use of genetic engineering for lignin reduction in plants and provides information about the agronomical and physiological suitability of *pPLAIIIδ* transgenic plants for utilization in biomass processing industries.

## 1 Introduction

Secondary wall growth is an important developmental feature in plants. It provides the plants with the needed mechanical strength for vertical growth extension. Secondary walls are characterized by the lignification of vascular tissues, which help plants to resist the negative pressure resulting from transpiration and endow the plants with rigidity and hydrophobicity (Zhong and Ye, 2015). However, the presence of lignin affects the utilization of plant biomass in various industries. The separation of cellulose from lignin during the production of bio-products such as bioethanol, textiles, and paper requires mechanical or chemical pretreatment processes, which are costly. Moreover, the methods used to remove the lignin can affect the quality and commercial value of the final bio-product (Liu et al., 2017). Plants that accumulate less lignin would therefore be ideal in bioenergy industries, because using such plants would reduce production costs and increase the quality of the manufactured bio-products. Several research groups have explored the use of molecular techniques as an alternative approach for reducing the lignin content of plants. For example, the mutation of two genes in the lignin biosynthesis pathway, caffeic acid 3-*O*-methyltransferase (COMT) and caffeoyl-CoA 3-*O*-methyltransferase (CCoAOMT), reduced the lignin biosynthesis yield in *Arabidopsis* (Xie et al., 2019). In another study, the downregulation of genes coding for cinnamyl alcohol dehydrogenase in *Morus alba* (*MaCADs*) reduced lignification in stems and leaves (Chao et al., 2022). Similarly, our group has reported the involvement of several members of group III patatin-related phospholipase genes (*pPLAIIIs*) during lignin biosynthesis processes (Jang et al., 2019; Jang and Lee, 2020a; Jang et al., 2022). We have reported that the independent overexpression of *pPLAIIIα* and *pPLAIIIγ* in *Arabidopsis* caused a significant decrease in lignin content (Jang and Lee, 2020a; Jang et al., 2022). Moreover, a decrease in lignin accumulation was observed when a homolog from *Panax ginseng, PgpPLAIIIβ*, was overexpressed in poplar and *Arabidopsis* (Jang et al., 2019; Jang and Lee, 2020b). The discovery of the role played by other members of the *pPLAIII* group in plant lignin biosynthesis prompted a study to determine if the remaining member of the group, *pPLAIIIδ*, also influences lignification. The *Arabidopsis pPLAIIIδ* is closely related to the rest members of *pPLAIII* group. Apart from hydrolyzing phospho/galactolipids, pPLAIII enzymes (pPLAIIIα, β, γ, and δ) are also involved in plant responses to abiotic stress, auxin signaling (Scherer et al., 2010), and in resistance to viral diseases (Jang et al., 2020). Also, pPLAIIIs influence the development of vegetative tissues and seeds (Li et al., 2011; Liu et al., 2015; Jang et al., 2021). Previous studies have indicated that *pPLAIIIδ* is specifically involved in several cellular functions, including growth, organ development, and signal transduction (Li et al., 2013; Dong et al., 2014; Li et al., 2015). For example, the overexpression of *pPLAIIIδ* was reported to enhance oil content and cause a dwarf phenotype in *Arabidopsis*, *Camelina sativa*, and *Brassica napus* (Li et al., 2013; Dong et al., 2014; Li et al., 2015). Additionally, *pPLAIIIδ* was reported to cause a decrease in cellulose content when overexpressed in *C. sativa* (Li et al., 2015). However, no study has reported the role played by *pPLAIIIδ* in the lignification of plant secondary walls. In this study, we found that the overexpression of *pPLAIIIδ* in *Arabidopsis* resulted in a significant decrease in lignification, while the silencing of *pPLAIIIδ* caused an increase in the lignin content. We also found that *pPLAIIIδ* played a significant role in plant growth, chlorophyll production, and its overexpression decreased the initial seed germination rate.

## 2 Materials and methods

### 2.1 Plant materials

The *Arabidopsis thaliana* Colombia ecotype (Col-0) was used as a wild type (WT) and background for the *pPLAIIIδ* gene overexpression and silenced lines. The *pplalllδ* (SALK_029470 and SALK_105929) SALK T-DNA insertion lines were obtained from the *Arabidopsis* Biological Resource Center (https://abrc.osu.edu/). *Arabidopsis* seeds were sown in half-strength Murashige and Skoog (MS) medium (Duchefa Biochemie, Haarlem, The Netherlands) containing 1% sucrose, 0.5 g/L 2-(N-morpholino) ethanesulfonic acid, 0.8% phytoagar, and the pH was adjusted to pH 5.7. Vernalization treatment was conducted for 2 days at 4°C under dark conditions, after which seeds were grown under long-day light (16 h light/8 h dark) at 23°C. *Arabidopsis* seedlings grown *in vitro* for 8 days were transferred to sterilized soil mixed with vermiculite and perlite at a 3:2:1 ratio.

### 2.2 Transgenic construction and *in planta* transformation

For the overexpression of *pPLAIIIδ* (*pPLAIIIδ-OE*), the full-length genomic sequence of *pPLAIIIδ* was obtained from wild-type *Arabidopsis* (Col-0) by polymerase chain reaction (PCR) using primers listed in **Supplementary Table 1**. Enzyme-digested PCR products were then cloned into the pCAMBIA 1390 vector driven by a native *PLAIIIδ* promoter and tagged with an enhanced cyan fluorescence protein at the C-terminus. To generate the *promoterPLAIIIδ::GUS* fusion construct (*PrompPLAIIIδ::GUS)*, a *pPLAIIIδ* full promoter region was amplified from the upstream intergenic region using primers listed in **Supplementary Table 1**. The PCR-amplified product was cloned into a vector pCAMBIA1390 which contained a *gusA* reporter gene. RNA interference (RNAi) was used to silence the *pPLAIIIδ* gene. A *pPLAIIIδ:RNAi* construct was generated through the amplification of a 164 bp of *pPLAIIIδ* gene coding sequence (sense) and its complementary sequence (antisense) using primers listed in **Supplementary Table 1**. The amplicons were cloned into the pHANNIBAL vector at XhoI/KpnI restriction enzyme sites (sense orientation) and HindIII/XbaI restriction enzyme sites (antisense orientation). The dsRNA cassette under the 35S cauliflower mosaic virus promoter was then recloned into the pART27 vector. The generated constructs were introduced into *Agrobacterium tumefaciens* C58C1 (pMP90), which were later transformed into *Arabidopsis* by the floral dipping method (Clough and Bent, 1998).

### 2.3 β-glucuronidase histochemical analysis

The β-glucuronidase (GUS) activity was visualized in *PrompPLAIIIδ::GUS* expressing transgenic plants by incubating them in the staining buffer containing 1 mM 5-bromo-4-chloro-3-indolyl-β-D-glucuronic acid cyclohexylammonium salt (X-Gluc, Duchefa Biochemie, Haarlem, The Netherlands), 0.1 M NaH_2_PO_4_, 0.01 M EDTA, 0.1% Triton-X, and 0.5 mM potassium ferri- and ferrocyanide in the dark at 37°C until blue color appeared. The stained plants were then washed in 70% (v/v) and 100% (v/v) ethanol sequentially for 2 hours each. Then, the plants were treated with a mixture of 10% (v/v) glycerol/50% (v/v) ethanol and 30% (v/v) glycerol/30% (v/v) ethanol to completely decolorize all non-stained tissues. A digital single-lens reflex (DSLR) camera (D8, Nikon, Tokyo, Japan) and a microscope (M165FC and DM3000 LED, Leica, Wetzlar, Germany) were used for imaging the GUS-stained plants.

### 2.4 Total RNA extraction and real-time quantitative PCR

*Arabidopsis* tissues from Col-0, vector control, *pPLAIIIδOE*, and *pPLAIIIδ:RNAi* were used for total RNA extraction following the TaKaRa MiniBEST plant RNA extraction kit (Takara, Shiga, Japan) user manual. The extracted total RNA was quantified using an ultraviolet (UV) spectrophotometer (Nano-MD, Scinco, Seoul, Korea). A 20 μL reaction volume was used to synthesize the complementary DNA (cDNA) using RevertAid reverse transcriptase (Thermo, Waltham, MA, USA). Quantitative PCR (qPCR) was conducted using TB Green™ Premix Ex Taq™ (Takara, Shiga, Japan) and a Thermal Cycler Dice real-time PCR system (Takara, Shiga, Japan). This was followed by standardization of the derived C_t_ value using the C_t_ value of a house-keeping gene *β-actin.* The expression level of the target gene was calculated through comparison with the control value using the formula 2^-ΔΔCt^. The specific primer sequences of the target genes analyzed by qPCR are listed in **Supplementary Table 1**.

### 2.5 Histochemical staining of lignin using phloroglucinol-HCl

Stem cross sections of 6-week-old Col-0, vector control, *pPLAIIIδ-OE*, and *pPLAIIIδ:RNAi* plants were used for lignin visualization. A sharp razor blade was used to cut 5 mm-thick sections from the stem taken at distance between 5 cm (basal sections) and 10 cm (apical sections) from the ground. The sections were treated with saturated phloroglucinol in HCl and immediately visualized and imaged under a microscope (M165FC and DM3000 LED, Leica, Wetzlar, Germany).

### 2.6 Acetyl bromide soluble lignin assay for total lignin quantification

The lignin content was determined using an acetyl bromide assay (Yan et al., 2012). Stem segments from 6-week-old plants were ground into fine powder in liquid nitrogen and then freeze-dried for 48 hours. Approximately 10 mg of powder was rinsed with 95% ethanol four times and twice with distilled water. The samples were then dried at 60°C after which they were suspended in 2 mL of 25% acetylbromide (v/v in glacial acetic acid). The samples were then incubated for 30 minutes at 70°C, followed by the addition of 0.9 mL of 2 M NaOH, 3 mL glacial acetic, and 0.1 mL 7.5 M hydroxylamine hydrochloride. The samples were centrifuged at 4000 g for 10 minutes, and the supernatant was diluted 20-fold with glacial acetic acid. Finally, the absorbance was determined at 280 nm using a UV spectrophotometer (Scinco, Seoul, Korea).

### 2.7 Chlorophyll and carotenoid content analysis

For chlorophylls extraction, rosette leaf samples taken from 4-week-old plants were ground in liquid nitrogen, and 50 mg of each sample was then mixed with 5 mL of 80% acetone. The mixture was well mixed and centrifuged at 3000 g for 10 minutes. The supernatant was pooled and measured for absorbance at 480, 663, and 650 nm using a Nano-MD UV–vis spectrophotometer (Scinco, Seoul, Korea). The chlorophyll and carotenoid contents were calculated using the standard equations (Wintermans and De Mots, 1965). The experiment was repeated at least three times.

### 2.8 Germination test

*Arabidopsis* seeds were sterilized using 70% ethanol and washed three times with autoclaved distilled water. The seeds were then sown and considered germinated when a radicle completely sprouted from a seed. Germinated seeds were counted at 20, 24, and 30 hours after exposure to light in the growth chamber. To analyze the effect of exogenous abscisic acid (ABA) on seed germination, 100 seeds (in triplicate) were sown in 1/2 MS media containing 0.1 and 0.5 μM ABA (Sigma-Aldrich, USA). The percent of germinated seeds was calculated after 24, 36, and 48 hours.

### 2.9 Lipid extraction and Lipidomic analysis

The lipid content of rosette leaves of 3-week-old plants was extracted. Lipidomic analysis was conducted via ultra-performance liquid chromatography (UPLC)-tandem mass spectrometry using an Ultimate RS 3000 UPLC system (Dionex) connected to a quadrupole-time-of-flight 5600 mass spectrometer (AB Sciex). Lipid extraction and lipidomic analysis procedures were conducted following the methods described by Jang et al. (2020).

## 3 Results

### 3.1 The expression characteristics of *pPLAIIIδ*

**Figure 1:**
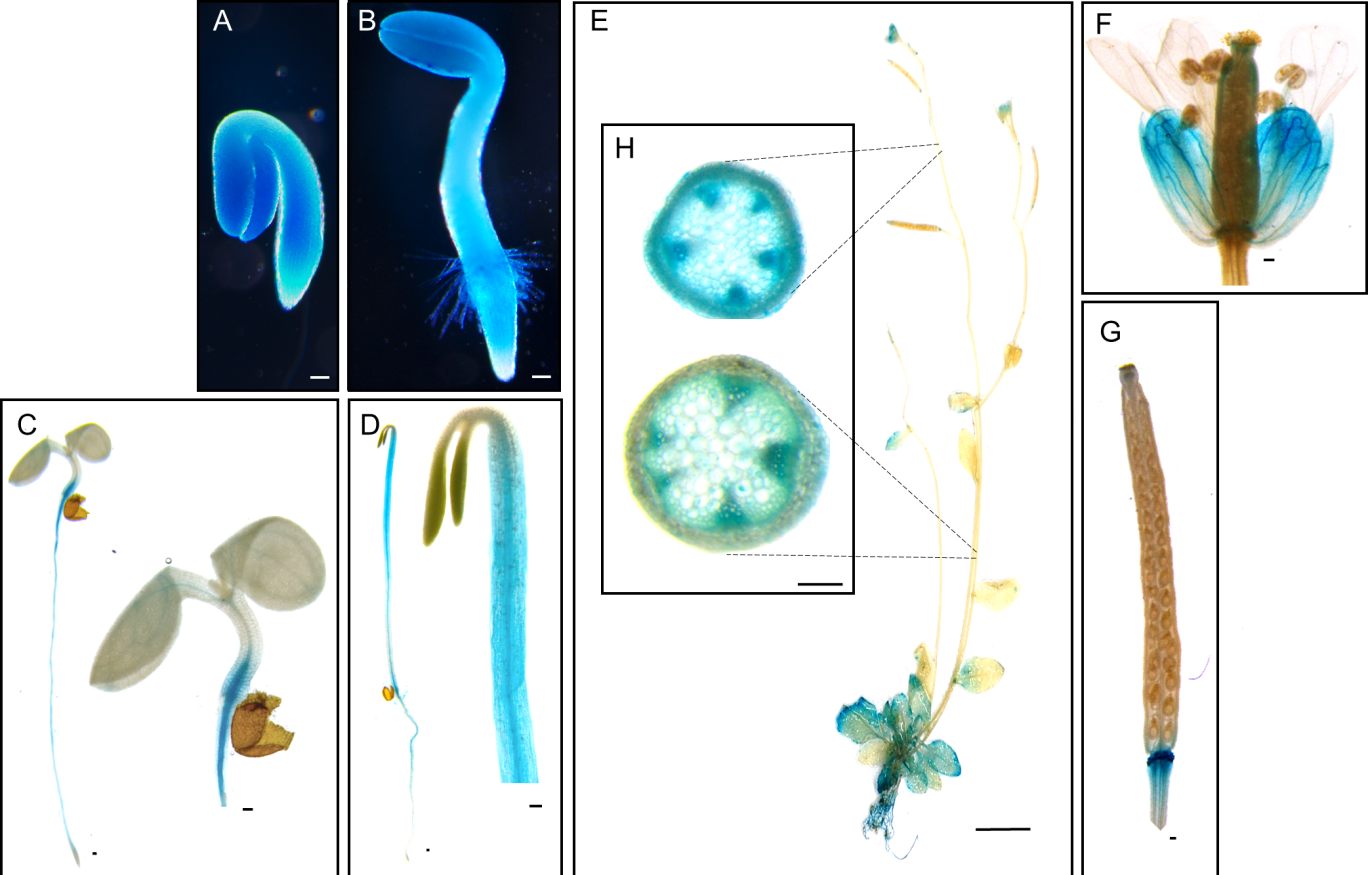
Histochemical analysis of GUS expression harboring *PrompPLAIIIδ::GUS* in different tissues at different stages of development. **(A)** 1-day-old seedling, **(B)** 2-day-old seedling, **(C)** 4-day-old seedling grown under long-day (16 h light/8 h dark) conditions, and **(D)** 4-day-old seedling grown under dark conditions. **(E)** 5-week-old plant. **(F)** Floral parts, and **(G)** silique. **(H)** Cross-sectional image of the apical and basal parts of the stem showing GUS expression in vascular bundles. Scale bars = 100 μm **(A–D, F–H)** and 1 cm **(E)**.

The *Arabidopsis pPLAIIIδ* is closely related to the rest members of group III *pPLAs* (Li et al., 2011; Jang et al., 2019; Jang and Lee, 2020a; Jang and Lee, 2020b; Jang et al., 2020). Its amino acid sequence shares the key conserved domains found in other pPLAIIIs. Among four isoforms of *pPLAIIIs*, *promoter::GUS* constructs of *pPLAIIIα* and *pPLAIIIγ* were expressed highly in xylem and phloem cells (Jang et al., 2020; Jang et al., 2022), and their overexpression under the 35S promoter reduced cell lignification (Jang and Lee, 2020a; Jang et al., 2022). To determine the functional redundancy of *pPLAIIIδ* with other homologs, *PrompPLAIIIδ::GUS* expression was studied. Our study aimed to elucidate where *pPLAIIIδ* is expressed, especially within tissues such as the stem vascular bundles, for which *pPLAIIIδ* expression has not yet been studied. Our results showed *pPLAIIIδ* expressed throughout a young sprouting seedling with a stronger GUS signal in the cotyledons (**Figures 1A, B**). The seedlings grown under long-day light conditions (**Figure 1C**) exhibited a different *pPLAIIIδ* expression distribution from those grown under dark conditions; the distribution became stronger in the hypocotyls of etiolated seedlings grown under dark conditions (**Figure 1D**). In a mature plant, *pPLAIIIδ* was highly expressed in roots and rosette leaves, while cauline leaves, siliques, and inner floral parts exhibited weaker GUS signals (**Figures 1E–G**). Furthermore, the cross sections of the apical and basal parts of the stem showed high expression in their vascular bundles (**Figure 1H**).

### 3.2 The effects on plant organ size caused by *pPLAIIIδ* overexpression and silencing

**Figure 2:**
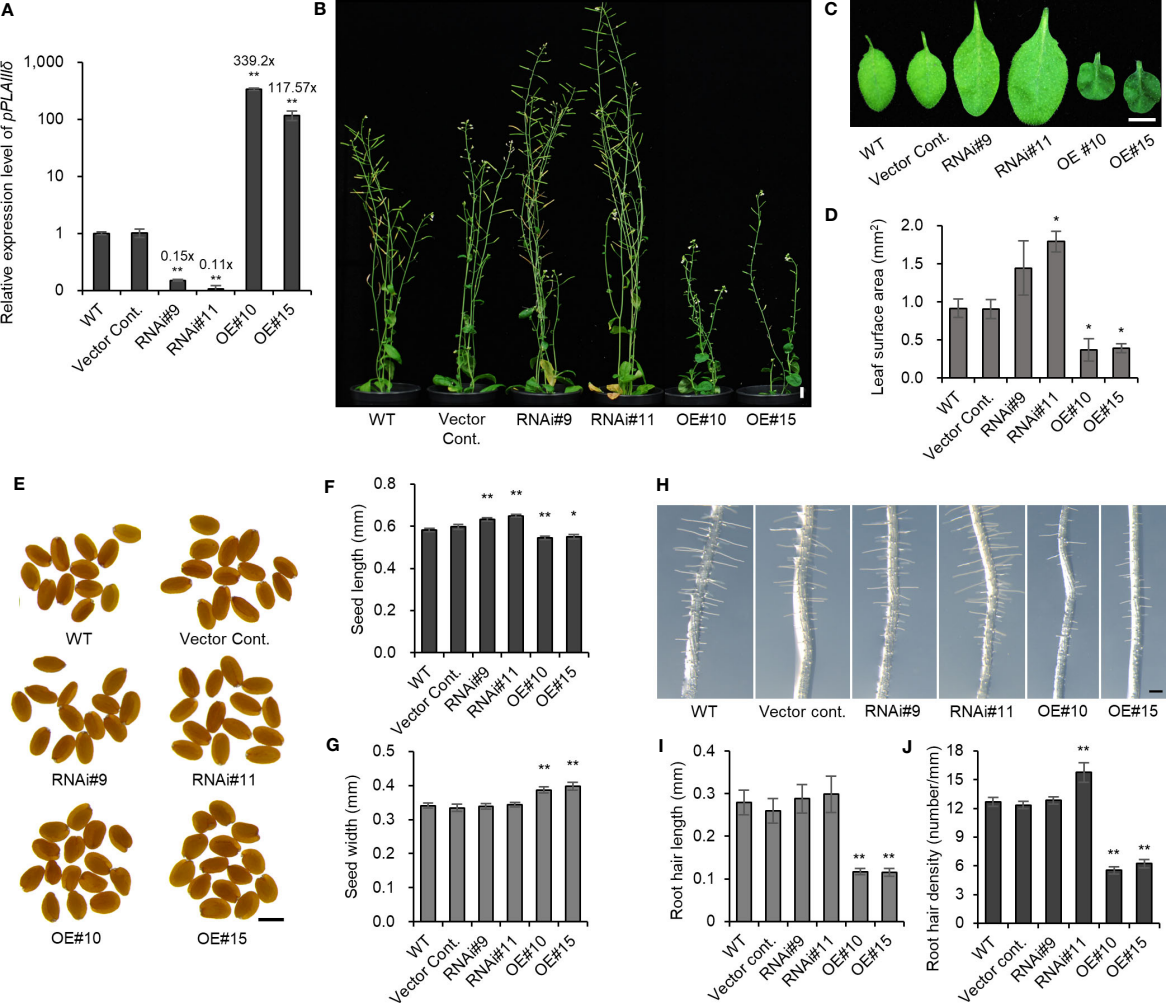
Overexpression of *pPLAIIIδ* alters the growth of plants. **(A)** *pPLAIIIδ* expression level in transgenic lines and in the controls. **(B)** 8-week-old plants. **(C)** Leaf phenotype and **(D)** leaf surface area. **(E)** Seed phenotype and statistical analysis of **(F)** seed length and **(G)** seed width. **(H)** 4-day-old root hair phenotype and quantification of **(I)** root hair length and **(J)** root hair density. Mean ± SE of three independent replicates. Asterisks indicate significant differences, obtained using the Student’s *t*-test (*P < 0.05 and **P < 0.01) compared with the wild type. Scale bar = 1 cm **(B, C)**, 500 μm **(E)**, and 0.2 mm **(H)**. WT, wild type; Vector Cont., vector control; RNAi, *pPLAIIIδ:RNAi*; OE, *pPLAIIIδ-OE*.

Studies have reported that the overexpression (*OE*) of *pPLAIII* members (*α, β, γ, δ*) caused a dwarf phenotype under the 35S promoter (Li et al., 2011; Li et al., 2013; Dong et al., 2014; Liu et al., 2015; Jang and Lee, 2020a; Jang and Lee, 2020b; Jang et al., 2020; Jang et al., 2022). A study using a T-DNA insertional *pplalllδ* mutant (SALK_029470) revealed few phenotypic differences (Li et al., 2013; Dong et al., 2014), because T-DNA insertions were not within exon, and the mutant was not a knockout (Li et al., 2013, **Supplementary Figure 1**). Another available T-DNA insertional *pplalllδ* mutant (SALK_105929) was also found to be an incomplete knockout (**Supplementary Figure 1**). For this reason, RNA interference (*pPLAIIIδ:RNAi*) was constructed to silence the *pPLAIIIδ* gene, which resulted in an average of 0.13× downregulation (**Figure 2A**; **Supplementary Figure 1**). The *pPLAIIIδ:RNAi* lines showed a significant increase in plant height, leaf surface area, and seed length (**Figures 2B–F**; **Supplementary Figure 2A**). However, the growth of the overexpression plants of *pPLAIIIδ* under native promoter (*pPLAIIIδ-OE*) was stunted, with reduced leaf surface area, reduced seed length, and increased seed width (**Figures 2B–G**). Moreover, *pPLAIIIδ-OE* lines showed shorter root hairs and reduced root hair density (**Figures 2H–J**). The root hair length was not significantly changed in *pPLAIIIδ:RNAi* lines, but root hair density increased in *pPLAIIIδ:RNAi*#11 (**Figures 2H–J**). The observed phenotypic results support previously reported effect of *pPLAIII* genes *OE* in impeding anisotropic cell expansion (Li et al., 2011; Jang and Lee, 2020a; Jang and Lee, 2020b; Jang et al., 2020; Jang et al., 2021; Jang et al., 2022). The decreased size of *pPLAIIIδ-OE* plants coincided with the downregulation of genes regulating active gibberellic acid (GA) biosynthesis, *GA20ox1* and *GA3ox1* (**Supplementary Figure 2B**). Expression of a GA catabolism gene *GA2ox1* was increased in *OE* line #10. In contrast, *GA2ox1* was downregulated in *pPLAIIIδ:RNAi* #11 (**Supplementary Figure 2C**). Another GA catabolism gene *GA2ox2* was unchanged in all transgenic lines. Surprisingly, the *GA3ox1* gene was downregulated in *pPLAIIIδ:RNAi* lines. Similar observations were reported in *OspPLAIIIα*-*OE* which caused dwarf rice plants by decreasing gibberellic acid sensitivity (Liu et al., 2015).

### 3.3 Alternation of lignin in *pPLAIIIδ-OE* transgenic *Arabidopsis*

**Figure 3:**
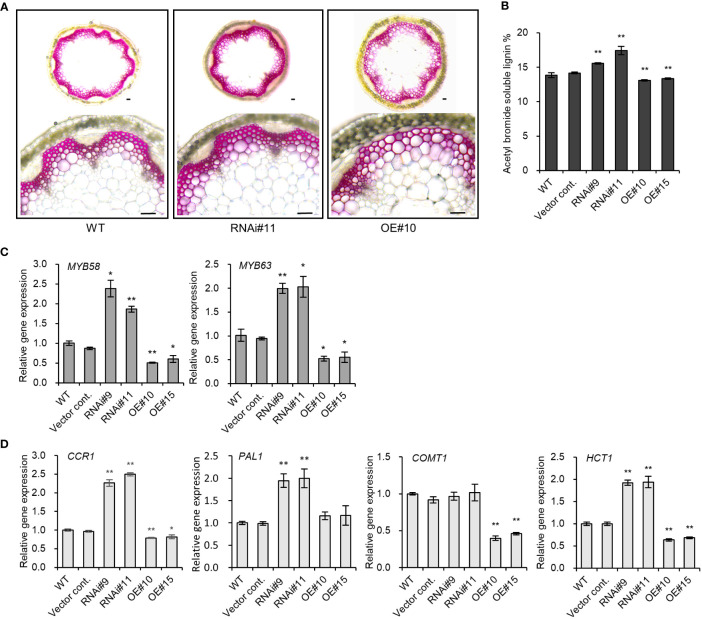
*pPLAIIIδ* alters the lignification of secondary walls. **(A)** Cross section of phloroglucinol-HCl stained *Arabidopsis* stem. Scale bar = 50 μm. **(B)** Quantification of the acetyl bromide soluble lignin content in RNAi, OE, and controls. **(C)** Transcription factors involved in the lignin biosynthesis pathway, and **(D)** the expression level of structural genes quantified by qPCR. Mean ± SE of the three independent replicates. Asterisks indicate significant differences, obtained using the Student’s *t*-test (*P < 0.05 and **P < 0.01) compared with the wild type.

The discovery of the role played by other members of the *pPLAIII* family in plant lignin biosynthesis prompted a study to determine if *pPLAIIIδ* also influences lignification. The expression of *PrompPLAIIIδ::GUS* in stem vascular cells (**Figure 1**) also indicates the possible involvement of *pPLAIIIδ* in lignin biosynthesis. Phloroglucinol-HCl staining is often used to visualize cell wall lignification as it can react with cinnamyl aldehydes to yield a pink or red-brown coloration (Pomer et al., 2002). Phloroglucinol-HCl staining was stronger in *pPLAIIIδ:RNAi* lines and weaker in *pPLAIIIδ-OE* lines compared with the controls (**Figure 3A**). The phloroglucinol-HCl staining results were consistent with results of the direct quantification of soluble lignin, which showed that the lignin content was significantly increased in *pPLAIIIδ:RNAi* lines but decreased in *pPLAIIIδ-OE* lines (**Figure 3B**). To identify the underlining mechanism through which *pPLAIIIδ* influences lignification, we checked the expression level of key transcription factors involved in the regulation of lignin biosynthesis, namely *MYB58* and *MYB63* (Zhou et al., 2009). Both transcription factors were upregulated in *pPLAIIIδ:RNAi* lines and downregulated in *pPLAIIIδ-OE* lines (**Figure 3C**). These results suggest that *pPLAIIIδ* functions upstream of the two lignin transcription factors. The structural genes in lignin biosynthesis are directly regulated by *MYB58* and *MYB68*, mostly through interactions at the AC-rich elements (Zhou et al., 2009; Zhao and Dixon, 2011). Thus, the expression levels of phenylalanine ammonia-lyase (PAL), cinnamoyl CoA reductase (CCR), hydroxylcinnamoyl transferase (HCT), and caffeic acid 3-*O*-methyltransferase (COMT) structural genes involved in lignin biosynthesis (Zhao and Dixon, 2011) were analyzed (**Figure 3D**). The qPCR analysis revealed an increased expression of *CCR1* (2.4-fold), *PAL* (1.9-fold), and *HCT* (1.9-fold) in *pPLAIIIδ:RNAi* lines, whereas the expressions of the genes decreased significantly in *OE* lines, except for *PAL*. The expression of the *CCR1* gene in *OE* lines decreased by an average of 1.25-folds, whereas that of *COMT* decreased by 2.5-folds, and that of *HCT1* decreased by 1.5-folds. Taken together, these results reveal that *pPLAIIIδ* negatively regulates lignin biosynthesis by influencing the expressions of *MYB58* and *MYB68*, which consequently affects the structural gene expression in the lignin biosynthesis pathway.

### 3.4 *pPLAIIIδ-OE* delayed seed germination via inhibition of bioactive gibberellin-biosynthetic genes

**Figure 4:**
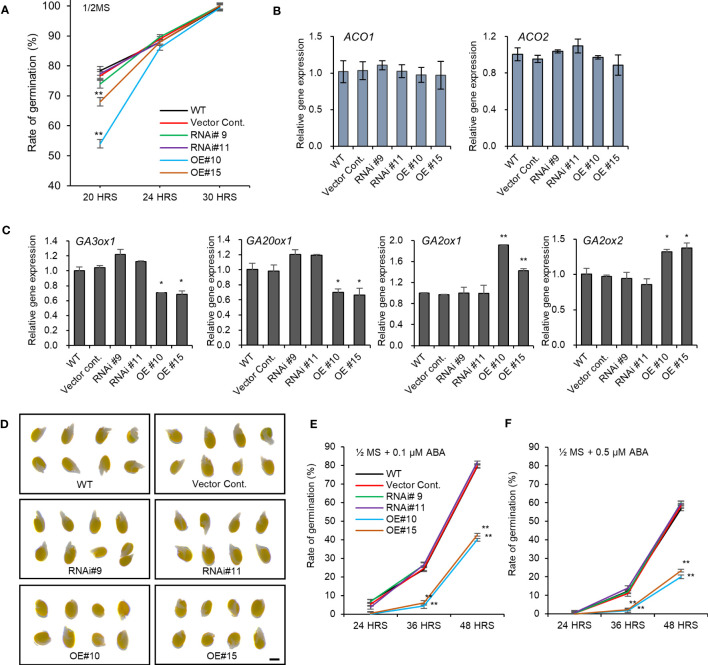
Seed germination rate altered by *pPLAIIIδ-OE.* **(A)** Seed germination rate. Expression levels of **(B)** ACC oxidases genes involved in ethylene biosynthesis. **(C)** Expression level of genes involved in active gibberellin biosynthesis (*GA3ox1, GA20ox1*), and genes involved in inactive gibberellin synthesis (*GA2ox1*, *GA2ox2*). **(D)** Seed germination with 0.1 μM ABA after 48 hours. **(E)** Seed germination rate with 0.1 μM ABA, and **(F)** 0.5 μM ABA. Mean ± SE of three independent replicates. Asterisks indicate significant differences, obtained using the Student’s *t*-test (*P < 0.05 and **P < 0.01). Scale bar= 0.5 mm.

The altered seed dimensions in *pPLAIIIδ* transgenic lines indicated a possible role of the gene in regulating seed germination. After 20 hours under light, the germination rate decreased by 24% in *pPLAIIIδ-OE* (**Figure 4A**). However, this initial delay in germination was not observed 30 hours after exposure to light because all seeds germinated (**Figure 4A**). *pPLAIIIδ:RNAi* showed no significant effect on the rate of seed germination. To confirm the delay of germination caused by *pPLAIIIδOE*, we used 24-hour imbibed seeds to check the expression level of genes involved in ethylene or gibberellin production. Studies have observed that seed germination depends on the accumulation of ethylene, which is produced by 1-aminocyclopropane-1-carboxylic acid (ACC) synthases (*ACS4*, *ACS5*, and *ACS11*) and oxidases (*ACO1* and *ACO2*) (Corbineau et al., 2014; Dong et al., 2014). The activities of *ACO1* and *ACO2* play a more significant role than the *ACS* activity during seed germination (Corbineau et al., 2014). No significant change in both the *ACO1* and *ACO2* expression levels occurred (**Figure 4B**). Three *ACS* genes were also not significantly changed compared with controls (**Supplementary Figure 3**). Thus, we checked if the expression level of the genes involved in gibberellic acid (GA) production changed in *pPLAIIIδ-OE* seeds (**Figure 4C**). GA plays an important role in improving germination by working antagonistically with abscisic acid (ABA), which is known as a germination inhibitor (Koornneef et al., 2002). The expression levels of *GA20ox1* and *GA3ox1*, which are the GA oxidases responsible for synthesizing bioactive gibberellins were downregulated 0.7-fold and 0.4-fold, respectively, in *pPLAIIIδ-OE.* Moreover, the expression levels of GA oxidases *GA2ox1* and *GA2ox2* in *pPLAIIIδ-OE* lines were increased, by 1.7- and 1.4-folds, respectively. *GA2ox1* and *GA2ox2* are known to inhibit active GA biosynthesis in plants (Li et al., 2021). The decrease of GA biosynthesis genes and increase of GA catabolism genes in *pPLAIIIδ-OE* germinating seeds, indicated an enhanced ABA sensitivity during seed germination. Germination test in media containing ABA showed *pPLAIIIδ-OE* seeds were more sensitive to ABA compared to the control seeds (**Figures 4D–F**). For example, the average germination of *pPLAIIIδ-OE* seeds in media containing 0.1 μM, and 0.5 μM ABA was reduced by 52.1%, and 38.2% respectively, 48 hours after exposure to light (**Figures 4E, F**). The germination rate of *pPLAIIIδ:RNAi* lines was not significantly changed. These results indicate that *pPLAIIIδ-OE* delays seed germination by affecting the ratio of GA to ABA.

### 3.5 *pPLAIIIδ-OE* regulates chlorophyll content by influencing MGDG production

**Figure 5:**
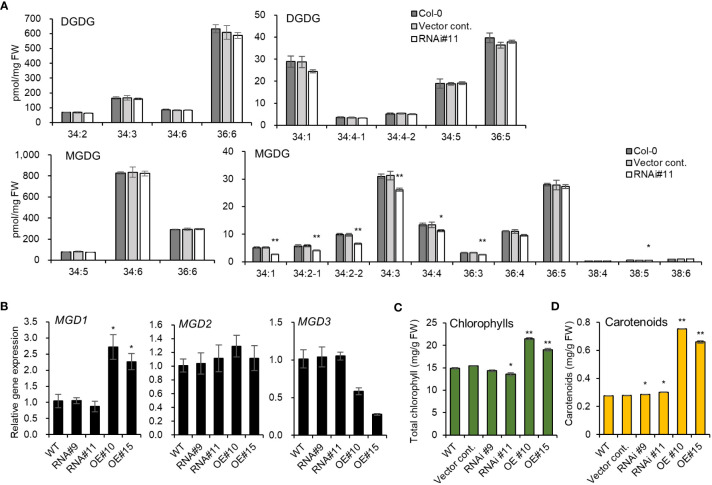
*pPLAIIIδ* plays a role in chlorophyll production. **(A)** The abundance of individual molecular species of galactolipids in *pPLAIIIδ:RNAi* and the controls. **(B)** The expression level of MGDG synthase genes, which codes for enzymes catalyzing MGDG synthesis. **(C)** Total chlorophylls, and **(D)** carotenoid contents. Mean ± SE of three independent replicates. Asterisks indicate significant differences, obtained using the Student’s *t*-test (*P < 0.05 and **P < 0.01) compared with the wild type.

The two galactolipids, monogalactosyldiacylglycerol (MGDG) and digalactosyldiacylglycerol (DGDG), constitute 75% of chloroplast lipidsthat play a key role in the structure and function of thylakoid membranes (Kobayashi et al., 2013). *pplaIIIδ* knockout mutant lines were reported to exhibit reduced total DGDG and MGDG levels compared to WT (Dong et al., 2014). Our study further identifies the individual molecular species of galactolipids that are affected by the silencing *pPLAIIIδ*, particularly because different molecular species may play different physiological roles (Wang et al., 2020). Using the *pPLAIIIδ:RNAi* line#11, we found that the significantly reduced MGDG molecular species were 34:1, 34:2-1, 34:2-2, 34:3, 34:4, 36:3, and 38:5 (**Figure 5A**). We did not find any significant difference in the amount of individual molecular species of DGDG (**Figure 5A**). To further elucidate the mechanism underlying the influence of *pPLAIIIδ* in MGDG production, we analyzed the expression levels of three MGDG synthase paralogs: *MGD1*, *MGD2*, and *MGD3. MGD1* is the major isoform involved in the synthesis of MGDG in chloroplasts, whereas *MGD2* and *MGD3* function to supply MGDG as a substrate for DGDG biosynthesis, and are activated during phosphate deficiency (Kobayashi et al., 2009). The expression level of *MGD1* was increased by 2.5-fold in *pPLAIIIδ-OE* lines, while the expression level of *MGD2* did not significantly change in all lines, and *MGD3* expression decreased by 0.3-fold in *pPLAIIIδ-OE* #10 (**Figure 5B**). The significant decrease in several molecular species of the chlorophyll-based lipid (MGDG) in *pPLAIIIδ:RNAi* and the increased expression of *MGD1* in *pPLAIIIδ-OE* lines indicated a functional role of *pPLAIIIδ* in chlorophyll synthesis and function. To prove this hypothesis, we analyzed the chlorophyll content in the transgenic lines. Quantification analysis revealed that *pPLAIIIδ-OE* lines had higher total chlorophyll and carotenoid contents than the controls (**Figures 5C, D**). Moreover, the total chlorophyll content decreased in *pPLAIIIδ:RNAi #*11, while the carotenoid level increased slightly in *pPLAIIIδ:RNAi* lines compared to the controls. The leaf phenotype of *pPLAIIIδ-OE* lines also showed a relatively darker greenish color compared with the color of the *pPLAIIIδ:RNAi* and control lines (**Figure 2C**). These results are similar to those found when a *PgpPLAIIIβ* was overexpressed in hybrid poplar plants (Jang et al., 2019), which signifies that *pPLAIIIs* play a key role in chlorophyll accumulation.

## 4 Discussion

Studying the overexpression and downregulation of *pPLAIIIδ* confirmed and elucidated the functional characteristics of the *pPLAIII* family genes. Specifically, the temporal and spatial expression patterns of *pPLAIIIδ* in roots and leaves were confirmed. In addition, the gene was well expressed in stem vascular bundles, where it played a significant role in secondary wall lignification. Our results also showed that the overall size of plants was reduced when *pPLAIIIδ* was overexpressed but enlarged when it was silenced. The altered seed size in *pPLAIIIδ-OE* led to delays in germination through the modulation of gibberellin-biosynthetic genes. This study also provides a link between *pPLAIIIδ*, MGDG levels, and chlorophyll contents.

### 4.1 Expression level of *pPLAIIIδ* influences the lignification of vascular tissues

The *pPLAIIIδ* expression was previously observed in roots, hypocotyl, the vascular bundles of leaves, and the meristem of the stem (Dong et al., 2014). Our study confirmed this expression pattern and also elucidated *pPLAIIIδ* expression in the stem xylem and phloem tissues (**Figure 1**). Expression in the vascular tissues of stems has also been observed in *pPLAIIIα* (Jang et al., 2020), and *pPLAIIIγ* (Jang et al., 2022). The two genes also played a role in the lignification of stem vascular tissues when overexpressed. Similarly, the phloroglucinol staining and the direct lignin quantification of stem tissues confirmed the role of *pPLAIIIδ* in stem lignification (**Figure 3**). Our results confirm the functional role of *pPLAIIIs* in plant lignification. Moreover, the increased lignin content in *pPLAIIIδ:RNAi* plants indicates that the gene played a more influential role in lignin formation than other members of the *pPLAIIIs*. In the case of *pplaIIIα* knockout mutants, the lignin level was not altered (Jang and Lee, 2020a). However, the downregulation or knockout effects of *pPLAIIIβ* and *pPLAIIIγ* genes on lignification needs to be studied further to confirm this notion.

### 4.2 The threshold level of *pPLAIIIδ* acts differently on plant growth

The overexpression of the *pPLAIII* members has been reported to cause stunted growth (Li et al., 2011; Dong et al., 2014; Jang and Lee, 2020a; Jang and Lee, 2020b; Jang et al., 2022), limiting their potential use in bio-products industries. However, the effects of *pPLAIIIs* downregulation on the size of plants have not been fully revealed. Thus far, only the *pplalllβ* mutant has been reported to influence plant growth by increasing leaf size and root length (Li et al., 2011). The knockout mutant of *pplalllα* did not show any phenotypic difference (Jang et al., 2020), while that of *pplalllγ* is yet to be identified (Jang et al., 2021). An incomplete knockout mutant of *pplalllδ* (SALK_029470) revealed few phenotypic differences such as longer hypocotyls and trichomes (Li et al., 2013; Dong et al., 2014). Owing to the lack of knockout mutants of the *pplalllδ* gene, this study generated the first RNAi silenced lines, which displayed larger leaves, taller plants, and longer seeds, with opposite results in *pPLAIIIδ-OE* (**Figure 2**). Overall, depending on the threshold level of the mRNA expression of *pPLAIIIs*, overexpression drives reduced longitudinal growth, while silencing promotes overall growth. The *pPLAIIIs*-influcenced growth alterations have been linked to several internal processes such as changes in macrotubule-associated proteins (Jang et al., 2021), and altered GA metabolism (Liu et al., 2015). This study has shown that decreased transcript level of GA biosynthesis genes could be another underlying mechanism for dwarf phenotype seen in OE lines. Generally, the increased size observed in *pPLAIIIδ:RNAi* lines, and reduced lignification reported in *pPLAIIIα and pPLAIIIγ-OE* indicate a possibility of co-regulation of *pPLAIII* genes to obtain a phenotypically suitable transgenic plant for bio-products industries.

### 4.3 Delayed germination in *pPLAIIIδ-OE* is mediated by increased transcripts involved in inactive GA biosynthesis

Although plant size and lignin amount are important traits in biomass processing industries, seed vigor is equally necessary for selecting suitable lines to be propagated for bioproducts manufacturing. Plants with rapid germination rate are considered more suitable, especially in the era of increased global temperatures (Reed et al., 2022). While the *pPLAIIIδ-OE* showed delayed seed germination, the *pPLAIIIα-OE* and the *PgpPLAIIIβ-OE* plants were reported to have increased the initial seed germination rate (Jang and Lee, 2020b; Jang et al., 2020). We further found that *pPLAIIIδ-OE* seeds were more sensitive to ABA (**Figures 4E, F**). The delayed germination in *pPLAIIIδ-OE* indicates that the gene behaves differently compared with *pPLAIIIα-OE* which increased seed germination by altering the biosynthesis of both ethylene and GA (Jang et al., 2020). These results suggest the existence of specific differential regulatory mechanisms in *pPLAIIIs*, underlined by changes in seed phenotype and initial germination rate. The antagonistic effects on the seed germination rate between *pPLAIIIδ-OE* and *pPLAIIIα-OE* suggest a possible gene suppression by ectopic gene expression. When we analyzed the transcript levels of the other *pPLAIII* genes in the *pPLAIIIδ* mutant lines (**Supplementary Figure 4**), the expression of *pPLAIIIα* was decreased in the *pPLAIIIδ-OE* lines, while the expression of *pPLAIIIγ* was increased in the *pPLAIIIδ:RNAi* lines. In the case of *pPLAIIIγOE*, the expression level of *pPLAIIIδ* was decreased (Jang et al., 2022), which also suggests the gene redundancy between *pPLAIIIγ* and *pPLAIIIδ*. Therefore, we can speculate that transgenic plants with silenced *pPLAIIIδ* and overexpressed *pPLAIIIα* could potentially have a good seed vigor as well as reduced lignin content making them suitable in biomass industries.

### 4.4 Decreased MGDG species affect the production of photosynthetic pigments

Silencing the *pPLAIIIδ* gene decreased key MGDG molecular species, including 34:3 (**Figure 5A**) which is required for photosynthesis (Wang et al., 2020), reducing the chlorophyll content (**Figure 5C**). Furthermore, the high level of photosynthetic pigments in the *pPLAIIIδ-OE* lines was a result of the increased expression of *MDG1* (**Figure 5B**). Similar to our results, overexpression of*pPLAIIIβ* increased chlorophylls content (Jang et al., 2019), and also increased the levels of MGDG and DGDG (Li et al., 2011). Moreover, similar to the effects of *pPLAIIIδ:RNAi* (**Figure 5A**), the *pplaIIIβ* and *pplalllδ* mutants also showed reduced galactolipids molecular species (Li et al., 2011; Dong et al., 2014). This suggests a functional redundancy between *pPLAIIIβ* and *pPLAIIIδ* during lipid biosynthesis. However, while silencing *pPLAIIIδ* resulted in reduced MGDG molecules (**Figure 5A**) and DGDGs (Dong et al., 2014), *pplaIIIα* knockout mutants did not change the levels of the two galactolipids (Jang et al., 2020). Nevertheless, the levels of MGDG and DGDG in *pPLAIIIα-OE* lines were decreased (Jang et al., 2020), indicating that another antagonistic function might exist between *pPLAIIIδ* and *pPLAIIIα*.

## Data availability statement

The original contributions presented in the study are included in the article/**Supplementary Material**, further inquiries can be directed to the corresponding author.

## Author contributions

OL conceived the project and designed the experiments. DS and JJ performed the experiments. OL, DS, and JJ analyzed the data and wrote the paper. All authors contributed to the article and approved the submitted version.
