# Supplementary material for: A group III patatin-like phospholipase gene pPLAIIIδ regulates lignin biosynthesis and influences the rate of seed germination in Arabidopsis thaliana
Source: Front Plant Sci. 2023 Jul 13;14:1212979. doi: 10.3389/fpls.2023.1212979 (PMC10372488; doi:10.3389/fpls.2023.1212979)
Supplement: Supplementary file 1 [file DataSheet_1.docx]

Supplementary Material

A group III patatin-like phospholipase gene pPLAIIIδ regulates lignin biosynthesis and influences the rate of seed germination in Arabidopsis thaliana

David Charles Simiyu, Jin Hoon Jang, and Ok Ran Lee*

*** Correspondence:** Ok Ran Lee (mpizlee@jnu.ac.kr)


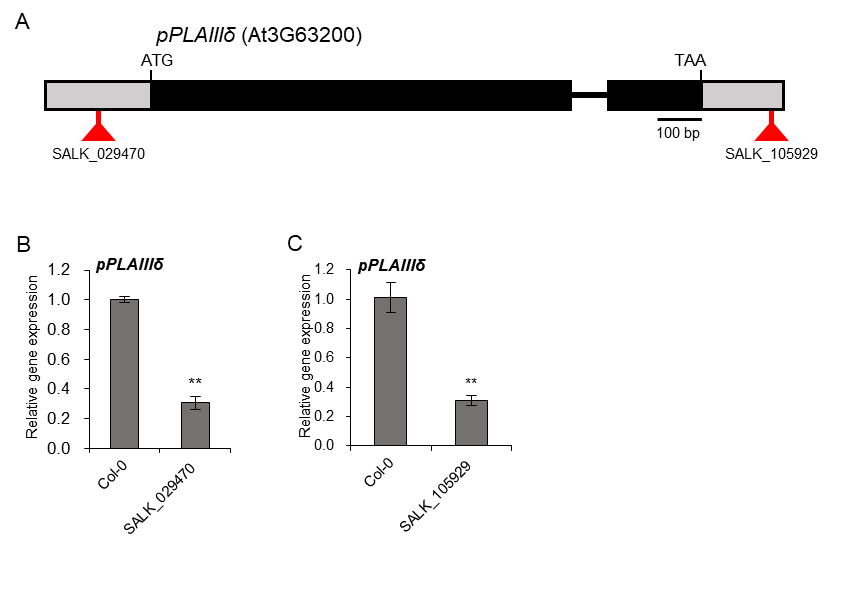


**Supplementary Figure S1.** Structure, and expression of *pPLAIIIδ* in SALK mutants. **(A)** *pPLAIIIδ* gene structure showing T-DNA insertion sites for SALK mutant lines. Gray area; intron, black area; exon. (**B, C**) Expression level of *pPLAIIIδ* in SALK mutant lines. Mean ± SE of three independent replicates. Asterisks indicate significant difference, obtained using the Student’s *t*-test (**P<0.01).


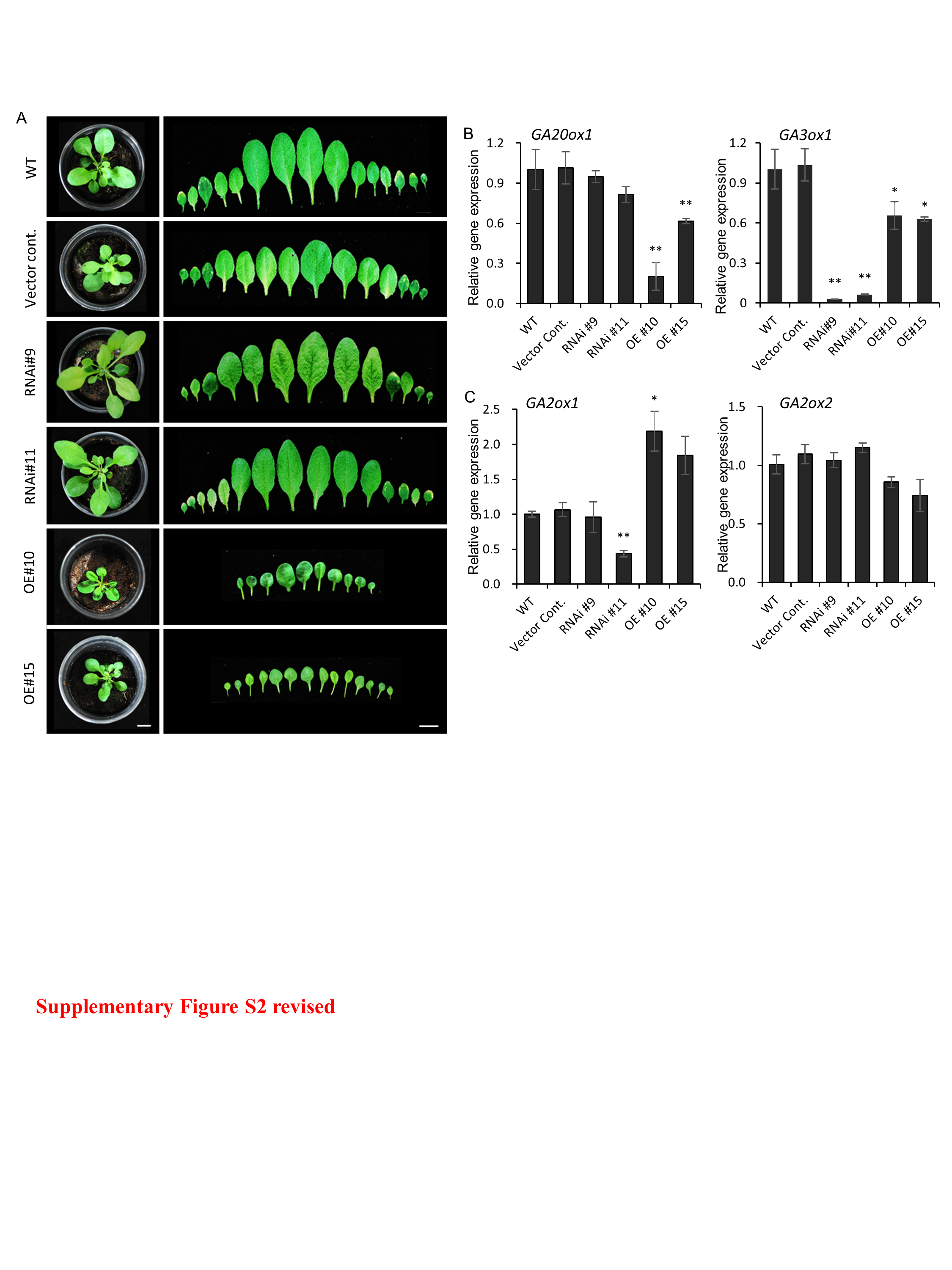


**Supplementary Figure S2.** *pPLAIIIδ* influence growth of plants and size of leaves. **(A)** Aerial parts of 4-weeks-old plants with their individual leaves. Expression level of **(B)** genes involved in active GA biosynthesis and **(C)** genes involved in GA catabolism. Mean ± SE of three independent replicates. Asterisks indicate significant difference, obtained using the Student’s *t*-test (*P<0.05 and **P<0.01). Scale bars=1 cm


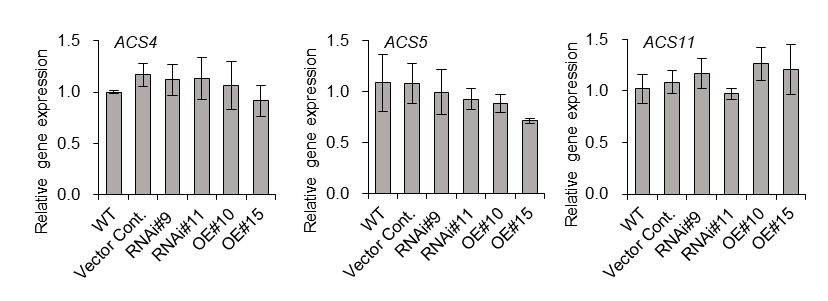
**Supplementary Figure S3.** Influence of *pPLAIIIδ* on ethylene biosynthesis genes expression during germination. Expression level of 1-aminocyclopropane-1-carboxylate synthases (*ACS*) genes in seeds imbibed for 24 hours. Mean ± SE of three independent replicates.


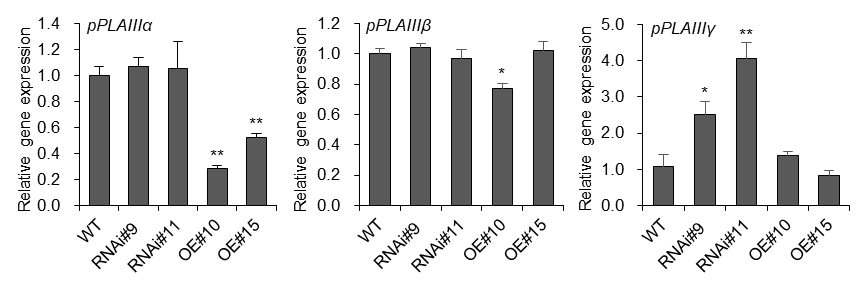


**Supplementary Figure S4.** Effect of differential mRNA levels of *pPLAIIIδ* on the expression of *pPLAIII* genes. Expression level of *pPLAIIIα, β, γ*, in *pPLAIIIδ-OE*, and *pPLAIIIδ:RNAi* lines as compared to the wild-type. Mean ± SE of three independent replicates. Asterisks indicate significant difference, obtained using the Student’s *t*-test (*P<0.05 and **P<0.01).

| **Supplementary Table 1. List of DNA primers used in this study** | | | |
| --- | --- | --- | --- |
| **Gene (AGI no.)** | **Annotation** | **Sequence** | **Purpose** |
| *pPLAIIIδ*  (At3g63200) | pPLAIIIδ:BamHI-5' | TCGGATCCCATGCCTACTCACACGTG | Cloning of *pPLAIIIδ: pPLAIIIδ-eCFP* |
|  | pPLAIIIδ:EcoRI-3' | TGGAATTCACGGCCGTCAGCGA |  |
|  | Prom_pPLAIIIδ:PstI-5' | TCCTGCAGGGACAGCTAACTATAATC | Cloning of *PrompPLAIIIδ::GUS* |
|  | Prom_ pPLAIIIδ:SaII-3' | TCGTCGACGCTGAGATCCATCTCCAT |  |
|  | RNAiIIId:XhoI-5' | ATCTCGAGTTCCTTGCTACGACCTCA | Cloning of *35S:pPLAIIIδ:RNAi* |
|  | RNAiIIId:KpnI-3' | AAGGTACCGGTTTTCCCGTCCACCGA |  |
|  | RNAiIIId:XbaI-5' | CGTCTAGATTCCTTGCTACGACCTCA |  |
|  | RNAiIIId:HindIII-3' | TTAAGCTTGGTTTTCCCGTCCACCGA |  |
|  | pPLAIIIδ-F | ACATTTTCACAAAGCTTGAACAGA | qRT-PCR |
|  | pPLAIIIδ-R | CCAAAACCCTCTCCATGCTCTTCC |  |
| *pPLAIIIα* | pPLAIIIα-F | GACGGATATGCAAGAACCGAGCAT |  |
|  | pPLAIIIα-R | TTCGAACCTGACCCACCCGAACCA |  |
| *pPLAIIIβ* | pPLAIIIβ-F | GCGGCTTCCAAGTCGGCGAATGAT |  |
|  | pPLAIIIβ-R | CTTAGCCGTCCCGGAACAACAACC |  |
| *pPLAIIIγ* | pPLAIIIγ-F | GTCCAACGTTATCAACGGCTCGAT |  |
|  | pPLAIIIγ-R | TCCTCCACTTCCGCTCCCTGATCT |  |
| *AtSAG12*  *(*At5g45890) | SAG12-5' | AATGATGAGCAAGCACTGATG |  |
|  | SAG12-3' | CGTAGTGCACTCTCCAGTGAA |  |
| *AtSAG13*  *(*At2g29350) | SAG13-5' | TTGCCCACCCATTGTTAAA |  |
|  | SAG13-3' | GATTCATGGCTCCTTTGGTT |  |
| *AtMGD1*  *(*At4g31780) | MGD1-5' | CATATGCAAAACCCTTCAACGGTAACC |  |
|  | MGD1-3' | GAATTCCCACAGAGTTCCATGTTTCACC |  |
| *AtMGD2*  *(*At5g20410) | MGD2-5' | AACATGTCTCCCTTAGTAGTTCTTTTGTC |  |
|  | MGD2-3' | GTTGATATTGTTAATGGCTAACAATAATGC |  |
| *AtMGD3*  *(*At2g11810) | MGD3-5' | GGATATCCATCATCTATCCCAACAA |  |
|  | MGD3-3' | GATAAAAGAATGACAAACCACTAGAGAATATAC |  |
| *AtACO1*  *(*At2g19590) | ACO1-5' | TGTCAGATCCCAAACATTTCAG |  |
|  | ACO1-3' | GGGTATTTAGCCACTTTTGTTCC |  |
| *AtACO2*  (At1g62380) | ACO2-5' | CGGGAAGTATAAGAGTGTGCTG |  |
|  | ACO2-3' | GGGTACTCGGAATCTTTCTCG |  |
| *AtGA20ox1*  *(*At4g25420) | GA20ox1-5' | CGGTTTCTTCCTCGTGGTCAATCA |  |
|  | GA20ox1-3' | CGTAGCCAACACTCTCACCGGATT |  |
| *AtGA3ox1*  (At1g15550) | GA3ox1-5' | ACCGACTCCACCCTCCTAACCATT |  |
|  | GA3ox1-3' | TAACCACGAGCGAGCCAGGAAA |  |
| *AtGA2ox1*  *(*At1g78440) | GA2ox1-5' | CCAGGTGATGACAAATGGGAGGTT |  |
|  | GA2ox1-3' | TCCTCGTACAACCTCTCGTCCTCATT |  |
| *AtGA2ox2*  *(*At1g30040) | GA2ox2-5' | GGTTTTGCCACAGCCAGTCACT |  |
|  | GA2ox2-3' | GGTTGACGACGGGGATTGAATGG |  |
| *AtMYB58*  (At1g16490) | MYB58-5' | CCAGAGAACAGAGCTCTTCAAGAG |  |
|  | MYB58-3' | ATGTATGAGGAGCTCGTAACTCTC |  |
| *AtMYB63*  (At1g79180) | MYB63-5' | GAACAGCTCAGGCTCAAGAGCAAC |  |
|  | MYB63-3' | ATGTATCATGAGCTCGTAGTTCTT |  |
| *AtCCR1*  (At1g15950) | CCR1-5' | TCCAGATGATCCGAAGAACA |  |
|  | CCR1-3' | CGCCTTAAGAGCCTCGTAGT |  |
| *AtPAL1*  *(*At2g37040) | PAL1-5' | CGCCTTAAGAGCCTCGTAGT |  |
|  | PAL1-3' | TCCTCGAAAGCTCCAATCTT |  |
| *AtCOMT*  *(*At1g21130) | COMT-5' | GTCGATTGCATTATGTTGGC |  |
|  | COMT-3' | AGCCTGATGCTTTGGCTAAT |  |
| *AtHCT*  *(*At5g48930) | HCT-5' | CTCTTTCCAAAGCCCTTGTC |  |
|  | HCT-3' | TCAGCCACAACGAAGAGAAC |  |
| *AtActin*  (At5g09810) | At-actin2-5' | GTGTGTCTTGTCTTATCTGGTTCG |  |
|  | At-actin2-3' | AATAGCTGCATTGTCACCCGATAC T |  |
